# Characterization of the complete chloroplast genome of *Aconitum flavum* (Ranunculaceae)

**DOI:** 10.1080/23802359.2020.1787894

**Published:** 2020-07-25

**Authors:** Yi Liu, Shuhua Yu, Fengming You

**Affiliations:** a Hospital of Chengdu University of Traditional Chinese Medicine, Chengdu, Sichuan, P. R. China; b College of Life Sciences, Sichuan Normal University, Chengdu, Sichuan, P. R. China

**Keywords:** *Aconitum flavum*, Ranunculaceae, Chinese medicine, chloroplast genome, phylogenetic tree

## Abstract

*Aconitum flavum*, a traditional Chinese medicine. The complete chloroplast genome sequence is 155,654 bp in length, with one large single copy region of 86,390 bp, one small single copy region of 16,968 bp, and two inverted repeat (IR) regions of 26,148 bp. It contains 129 genes, including 83 protein-coding genes, 8 ribosomal RNA, and 37 transfer RNA. Phylogenetic tree shows that this species is a sister to *A. brachypodum*.

The Aconitum consisting of over 200 species distributed in China, Most of them grow among high altitudes. Such as *A. kusnezoffii*, *A. carmichaelithe* and *A. flavum* (Xiao 2006). In traditional Chinese medicine, Aconitum have similar pharmacological actions and are commonly applied for various inflammatory diseases, such as rheumatic fever, painful joints, gastroenteritis, diarrhea, edema, bronchial asthma and various tumors (Singhuber et al. 2009; Qin et al. 2012). However, due to anthropogenic over exploitation and decreasing distributions, this species needs reagent conservation. Knowledge of the genetic information of this species would contribute to the formulation of protection strategy. In this study, we assembled and characterized the complete chloroplast (cp) genome sequence of *A. flavum*.

Fresh leaves of *A. flavum* were collected from Nanhuashan mountain (Zhongwei, Ningxia, China; coordinates: 105°36′E, 36°26′N) and dried with silica gel. The voucher specimen was stored in Sichuan University Herbarium with the accstion number of QTPLJQ13383066. Total genomic DNA was extracted with a modified CTAB method (Doyle and Doyle 1987). First, we obtained 10 million high quality pair-end reads for *A. flavum*, and after removing the adapters, the remained reads were used to assemble the complete chloroplast genome by NOVOPlasty (Dierckxsens et al. 2017). The complete chloroplasts genome sequence of *A. brachypodum* was used as a reference. Plann v1.1 (Huang and Cronk 2015) and Geneious v11.0.3 (Kearse et al. 2012) were used to annotate the chloroplasts genome and correct the annotation.

The total plastome length of *A. flavum* (MT571464) is 155,654 bp, exhibits a typical quadripartite structural organization, consisting of a large single copy (LSC) region of 86,390 bp, two inverted repeat (IR) regions of 26,148 bp and a small single copy (SSC) region of 16,968 bp. The cp genome contains 129 complete genes, including 83 protein-coding genes (83 PCGs), 8 ribosomal RNA genes (4 rRNAs), and 37 tRNA genes (37 tRNAs). Most genes occur in a single copy, while 13 genes occur in double, including 7 tRNAs (trnA-UGC, trnI-CAU, trnI-GAU, trnL-CAA, trnN-GUU, trnR-ACG, and trnV-GAC), and 6 PCGs (rps7, rpl2, rpl23, ndhB, ycf2, ycf15). The overall GC content of cp DNA is 38.1%, the corresponding values of the LSC, SSC, and IR regions are 36.2, 32.6, and 43.0%.

In order to further clarify the phylogenetic position of *A. flavum*, plastome of nine representative *Aconitum* species were obtained from NCBI to construct the plastome phylogeny, with *Delphinium anthriscifolium* as an outgroup. All the sequences were aligned using MAFFT v.7.313 (Katoh and Standley 2013) and maximum likelihood phylogenetic analyses were conducted using RAxML v.8.2.11 (Stamatakis 2014). The phylogenetic tree shows that *Aconitum* clade were identified as two subclades. *A. japonicum*, *A. jaluense* and *A. kusnezoffii* together one clustered. Remian *Aconitum* species together another clustered, While *Aconitum brachypodum* clustered together with *A. flavum* in this subclade (Figure 1).

**Figure 1.**
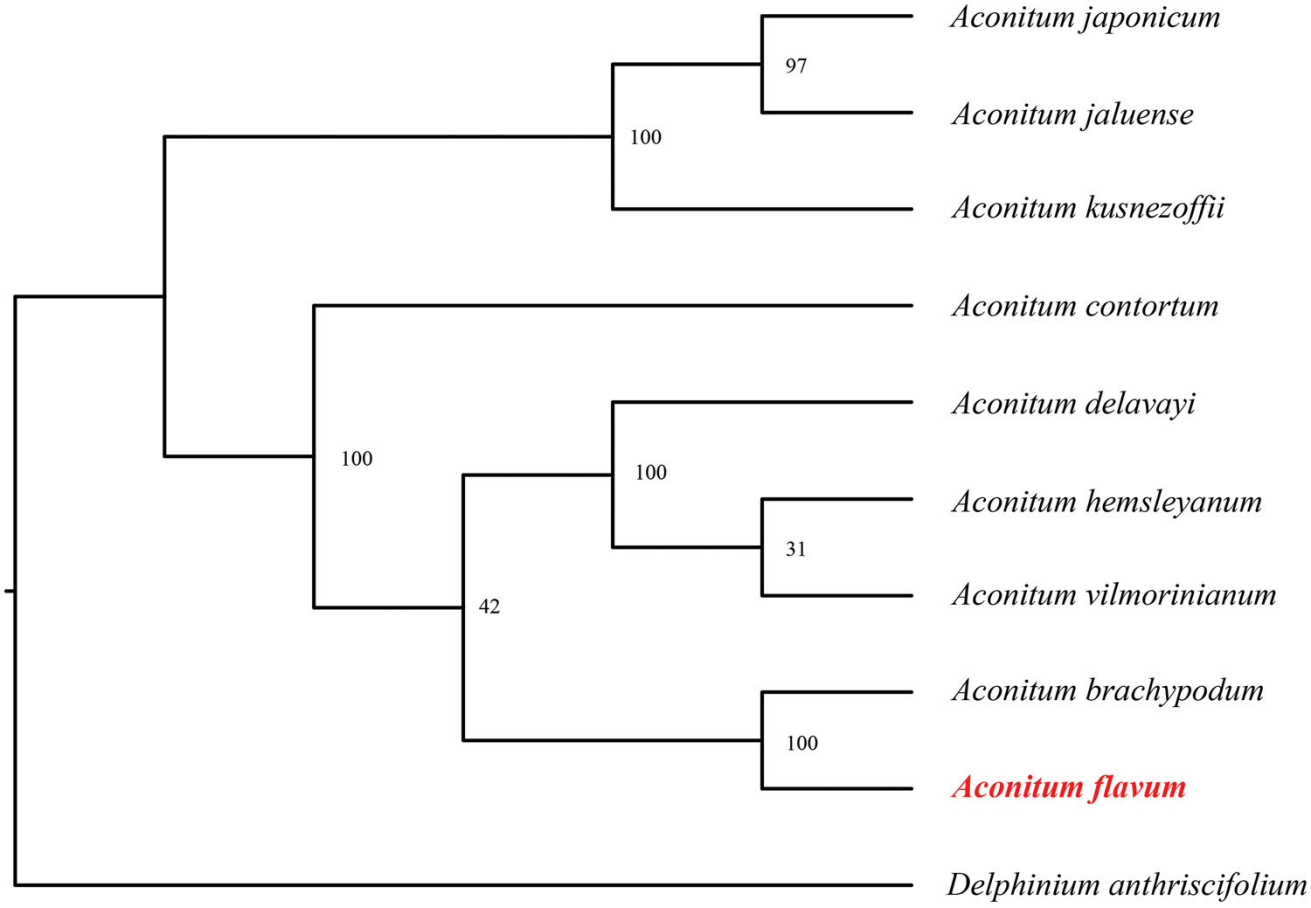
Phylogenetic relationships of *Aconitum* species using whole chloroplast genome. GenBank accession numbers: *Aconitum brachypodum* (NC_041579), *Aconitum contortum* (NC_038098), *Aconitum delavayi* (NC_038097), *Aconitum episcopale* (NC_038096), *Aconitum hemsleyanum* (NC_038095), *Aconitum jaluense* (KT820669), *Aconitum japonicum* (KT820670), *Aconitum kusnezoffii* (MK569468), *Aconitum vilmorinianum* (NC_038094), *Delphinium anthriscifolium* (MK253461).

## Data Availability

The data that support the findings of this study are openly available in GenBank of NCBI at https://www.ncbi.nlm.nih.gov, reference number MT571464.
